# Is tobacco dependence a moderator of psychiatric symptom severity and caregiver abuse in rural families of patients with severe mental disorders?

**DOI:** 10.1017/S0033291725102663

**Published:** 2025-11-28

**Authors:** Afei Qin, Meiqi Wang, Yazhuo Qi, Kaixian Wang, Zhen Wei, Long Sun

**Affiliations:** 1 Department of Social Medicine and Health Management, School of Public Health, Cheeloo College of Medicine, Shandong University, Jinan, China; 2 NHC Key Lab of Health Economics and Policy Research, Shandong University, Jinan, China; 3 Center for Health Management and Policy Research, Shandong University, Jinan, China; 4 School (Institute) of Mental Health and Psychological Sciences, Cheeloo College of Medicine, Shandong University, Jinan, China

**Keywords:** abuse, caregiver, psychiatric symptom severity, severe mental disorders, tobacco dependence

## Abstract

**Background:**

Severe mental disorders (SMDs) impose profound suffering on patients and heavy burdens on family caregivers, often resulting in abusive behaviors. This study aimed to examine the association between psychiatric symptom severity and caregiver abuse, and to assess whether caregiver tobacco dependence moderates this relationship.

**Methods:**

A cross-sectional study included 763 patient–caregiver dyads in rural Shandong, China. Psychiatric symptom severity was measured using the 18-item Brief Psychiatric Rating Scale. Caregiver tobacco dependence was assessed using the Fagerström Test for Nicotine Dependence. Patients reported caregivers’ verbal/physical abuse in the past year. Ordered logistic regression and interaction terms tested associations and moderation.

**Results:**

Overall, 25.7% of caregivers engaged in verbal abuse and 14.9% in physical abuse. Psychiatric symptom severity was significantly associated with both verbal (OR = 1.018, 95% CI: 1.010–1.026) and physical abuse (OR = 1.015, 95% CI: 1.005–1.025). Caregivers with moderate to severe tobacco dependence were more likely to commit verbal (OR = 1.851, 95% CI: 1.136–3.016) and physical abuse (OR = 2.292, 95% CI: 1.287–4.079) than non-smokers. Moderate to severe tobacco dependence significantly amplified the association between psychiatric symptom severity and verbal abuse (interaction OR = 1.024, 95% CI: 1.002–1.046), but not physical abuse.

**Conclusion:**

In rural China, greater psychiatric symptom severity among patients with SMDs is associated with increased frequency of both verbal and physical abuse by caregivers, particularly verbal abuse among those with moderate to severe tobacco dependence, underscoring the need for caregiver-targeted psychological support and tobacco cessation interventions.

## Introduction

Mental disorders are among the top 10 causes of global disease burden, with disability-adjusted life years attributable to them continuing to rise (GBD 2019 Mental Disorders Collaborators, 2022). Severe mental disorders (SMDs) not only impose profound suffering on patients but also place a substantial burden on family caregivers (Estrada-Fernández et al., 2022; Sin et al., 2021). Prolonged caregiving stress is strongly associated with impaired mental health and reduced quality of life among caregivers (Akbari et al., 2018; Estrada-Fernández et al., 2022; Oikonomou et al., 2024; Sin et al., 2021), and in some cases may result in abusive behaviors toward patients. Evidence indicates that people with mental disorders face a markedly higher risk of abuse compared with the general population (Dean et al., 2024; Marr et al., 2024). However, while a large body of research has examined caregiver abuse, most studies have focused on patients with dementia and older adults (Hernandez Chilatra et al., 2025; Pickering et al., 2024; Shen et al., 2025; Wei et al., 2024; Yon et al., 2017). Evidence concerning SMDs remains scarce. Family abuse not only violates patients’ rights and worsens clinical outcomes but may also reinforce a vicious cycle between mental illness and family dysfunction, underscoring the urgency of addressing this issue.

Psychiatric symptom severity, which constitutes the core diagnostic criteria of mental disorders, is closely linked with reduced quality of life and increased mortality (Chene et al., 2023; Zhou et al., 2024), and may also contribute to aggressive behaviors (Belete et al., 2016). Yet the direct associations and mechanisms linking psychiatric symptom severity in SMDs to caregiver abuse remain poorly understood. Dementia research suggests that neuropsychiatric symptom severity may mediate the relationship between patient condition and caregiver abuse (Fang et al., 2021), providing a conceptual foundation for exploring similar dynamics in SMDs.

In this process, caregivers’ own risk behaviors, such as tobacco dependence, may represent critical but overlooked factors. Chronic caregiving stress increases caregivers’ vulnerability to tobacco dependence, which exacerbates mental health problems through biopsychosocial pathways (Airagnes et al., 2025) and contributes to serious physical illness (Ahluwalia et al., 2018). According to stress–coping theory (Biggs et al., 2017), when environmental stressors exceed an individual’s coping resources, maladaptive behavioral responses may ensue. This framework offers insight into patterns of abusive caregiving. When psychiatric symptom severity in patients acts as stressors that surpass caregivers’ coping capacity, tobacco dependence may reflect depleted coping resources, thereby amplifying the risk of abuse. Strain theory (Zhang, 2019; Zhang et al., 2011) also explains how prolonged caregiving pressure and frustration build up into psychological strain. When caregivers repeatedly fail to meet expectations of effective care, such strain may trigger maladaptive coping behaviors, such as increased smoking or verbal aggression, as temporary means to reduce inner tension. Meanwhile, insights from self-control theory (Gottfredson & Hirschi, 1990) suggest that chronic stress and nicotine dependence can jointly impair emotional regulation and inhibitory control, thereby increasing impulsivity and aggressive responses. Together, these frameworks clarify the interlinkages between caregiving stress, tobacco dependence, and abusive behaviors in families of SMD patients.

In China, mental health resources remain severely constrained, with rural areas disproportionately affected (Sun et al., 2024; Xiang et al., 2018; Xiang et al., 2012). Beyond heavy economic burdens (Xu et al., 2016; Yan & Tu, 2022), rural caregivers often lack professional support, leaving them more vulnerable to distress and more likely to perpetrate abuse. Patients with mental disorders, due to their cognitive, emotional, and behavioral impairments, are highly dependent and marginalized, which makes them particularly vulnerable to hidden and harmful forms of abuse. Research has shown that family settings are the second most frequent sites of abuse, after public spaces (Drew et al., 2011). Although the World Health Organization has called for the protection of people with mental disorders from exploitation, violence, and abuse (World Health Organization, 2022), empirical evidence in rural China remains limited.

Against this backdrop, this study used paired data from patients with SMDs and their family caregivers in rural China to: (1) examine the associations of patients’ psychiatric symptom severity and caregivers’ tobacco dependence on abusive behaviors (verbal and physical abuse); and (2) assess whether tobacco dependence moderates the relationship between psychiatric symptom severity and caregiver abuse.

## Methods

### Study design, sampling, and data collection

This cross-sectional study was conducted in rural areas of Shandong Province, China, between August 2022 and August 2024. To ensure representativeness and capture variation across different levels of economic development, we employed a rigorous multistage stratified random sampling strategy. First, all 16 prefecture-level cities in Shandong were stratified into high and low economic tiers according to annual gross domestic product. One city was randomly selected from each tier: Jinan (high) and Zaozhuang (low). Within these, Zhangqiu District of Jinan and Taierzhuang District of Zaozhuang were randomly selected as study sites. Focusing on rural populations, six townships were randomly drawn from each district, resulting in a total of 12 study sites. All registered patients with SMDs in these areas – including schizophrenia, schizoaffective disorder, paranoid psychosis, bipolar disorder, epilepsy-related psychosis, and intellectual disability with psychiatric comorbidities – along with their primary caregivers, were identified as potential participants. The majority of caregivers were family members (e.g. spouses, children, or parents), while a small proportion were non-family caregivers, including the patient’s grandparents, maternal grandparents, other relatives, or friends.

Data were collected through one-to-one structured, face-to-face interviews. Given the specific challenges of interviewing people with SMDs in rural settings, interviews were conducted either at patients’ homes or in familiar community-based environments, such as village clinics, township health centers, community health service stations, or local party–mass service centers. A local family doctor was present throughout, and the patient’s primary caregiver was required to attend and participate. For patients with cognitive impairment or communication difficulties, responses were primarily provided by caregivers or another close relative familiar with the patient’s daily functioning. For patients with preserved communication ability, self-reports were encouraged, with caregivers offering supplementary assistance if necessary. Each questionnaire was independently cross-checked by at least two trained investigators to ensure consistency and completeness. In total, 809 patient–caregiver dyads were surveyed. After excluding 14 dyads where caregivers were younger than 16 years and 32 dyads with substantial missing information, 763 dyads were included in the final analysis.

The study adhered to the principles of the Declaration of Helsinki and relevant Chinese regulations on research involving human participants. Ethical approval was granted by the Ethics Committee of the School of Public Health, Shandong University (approval number: LL20210803). Before interviews, investigators provided detailed explanations of study objectives and procedures to patients and caregivers. Written informed consent was obtained from all participants or their legal guardians. For individuals unable to sign, oral consent was documented in the presence of an independent witness.

### Measurement method

#### Caregiver abuse reported by SMD patients

Abuse by caregivers in the past year was assessed through structured questions, covering two domains:

- Verbal abuse: patients were asked, ‘Have you ever been verbally abused by a caregiver (e.g. using derogatory, threatening, or demeaning language)?’ Response options: (1) Never; (2) Several times a year (at least once, but less than once a month); (3) Several times a month (at least once, but less than once a week); (4) Several times a week (at least once, but less than once a day); and (5) Once a day or more
- Physical abuse: patients were asked, ‘Have you ever been physically abused by a caregiver (e.g. pushing, slapping, punching, kicking, or other behaviors causing discomfort or injury)?’ Response options were identical to those above.

Responses were coded on a 5-point scale (1–5), with higher scores indicating greater frequency of abuse. Data were primarily based on patient self-report. For patients with severe cognitive impairment or communication difficulties, answers were provided by their main caregiver (not the alleged abuser) or another close relative based on observation.

#### Psychiatric symptom severity

Psychiatric symptom severity was measured using the 18-item Brief Psychiatric Rating Scale (BPRS-18) (Overall & Gorham, 1962). The BPRS-18 is a widely validated tool in psychiatric research and practice (Leucht et al., 2005; Tatsumi et al., 2020), covering domains such as thought disturbance, hostility–suspicion, activation, and anxiety–depression. Trained investigators rated each item based on the patient’s recent (typically past week) status, informed by both observation and interview. Each item was scored on a 7-point scale (1 = not present, 7 = extremely severe), yielding a total score range of 18–126, with higher scores indicating more severe symptoms. The Cronbach’s α coefficient of BPRS-18 in this study is 0.859.

#### Caregiver tobacco dependence

Caregiver tobacco dependence was assessed using the Fagerström Test for Nicotine Dependence (FTND) (Heatherton et al., 1991), a standardized and widely validated tool (Kozlowski et al., 1994). The FTND consists of six items addressing key features of nicotine dependence, including time to first cigarette after waking, daily cigarette consumption, and difficulty refraining from smoking. Item scores range from 0 to 1 or 0 to 3, yielding a total score of 0–10, with higher scores reflecting greater dependence. Current smoking status was first confirmed through a screening question; the FTND was then administered only to current smokers. Following established cut-offs (Liu et al., 2022; Quach et al., 2020), caregivers were categorized as non-smokers (screened negative), mild dependence (FTND score 0–3), or moderate-to-severe dependence (FTND score ≥4).

#### Measurement of covariates

We collected covariates related to the caregivers, including relationship with patients (categorized as spousal, the patient’s child, the patient’s parent, or others, including the patient’s grandparents, maternal grandparents, relatives or friends), gender (male/female), marital status (married/others, including never married, divorced and widowed), age in years (continuous), educational level (illiterate or semi-illiterate, primary school, junior high school, or high school and above), occupation (farmer, workplace professionals and students, unemployed, or others, including retirement and engaging in informal occupations), religious belief (no/yes), annual household income (divided into levels 1 to 4 based on quartile scores, with higher levels indicating higher income), caregiving duration (<1 year, 1–2 years, 2–3 years, or >3 years), daily average caregiving hours (continuous), provision of financial assistance (no/yes), and total household debt per 10 thousand RMB (continuous).

### Statistical analyses

Firstly, descriptive statistics were presented as frequencies (%) for categorical variables and mean ± SD for continuous variables. Group differences across abuse frequency categories were assessed using chi-square tests for categorical variables and one-way ANOVA for continuous variables. Second, ordered logistic regression was performed to test the effects of patient’s BPRS score and caregiver tobacco dependence on verbal/physical abuse frequency (5-level ordinal outcome), adjusting for all covariates. Thirdly, moderation analysis evaluated whether tobacco dependence modified BPRS-abuse relationships by adding BPRS score × tobacco dependence interaction terms to the models. Results are reported as adjusted odds ratios (OR) with 95% CIs. All analyses were conducted using StataMP 17.0. A two-sided *p*-value <0.05 was regarded as statistically significant.

## Results

### The basic characteristics of abusive behavior by caregivers

Table 1 presents the characteristics of caregivers and the distribution of verbal abuse. Among the 763 caregivers of patients with SMDs, approximately half were spouses of the patients (390, 51.1%), most were men (485, 63.6%), the majority were married (665, 87.2%), and the mean age was 59.9 years (SD 12.6). More than half had only primary education or below (447, 58.6%). A total of 196 caregivers (25.7%) reported verbal abuse toward patients. Among them, nine caregivers (1.2%) committed verbal abuse more than once per day, 47 (6.2%) more than once per week, 64 (8.4%) more than once per month, and 76 (10.0%) more than once per year. Higher BPRS scores in patients were significantly associated with greater frequency of verbal abuse (*p* < 0.001), as were higher levels of caregiver tobacco dependence (*p* < 0.001). Other variables associated with verbal abuse included annual household income, average daily caregiving time, receipt of social assistance, and total household debt (all *p* < 0.05).

**Table 1.** Frequency of caregiver verbal abuse toward SMDs patients (n (%)/mean ± SD)

Variables		Frequency of verbal abuse by caregivers toward patients					*F*/*χ^2^*
	Total	Never	Several times a year (at least once, but less than once a month)	Several times a month (at least once, but less than once a week)	Several times a week (at least once, but less than once a day)	Once a day or more	
Total	763 (100.00)	567 (74.31)	76 (9.96)	64 (8.39)	47 (6.16)	9 (1.18)	
BPRS score	51.09 ± 20.48	49.28 ± 19.69	51.09 ± 20.81	57.36 ± 22.75	60.60 ± 18.84	70.56 ± 28.98	7.41***
Tobacco dependence							30.00***
Non-smoking	554 (72.61)	427 (77.08)	45 (8.12)	46 (8.30)	34 (6.14)	2 (0.36)	
Mild tobacco dependence	92 (12.06)	61 (66.30)	17 (18.48)	7 (7.61)	6 (6.52)	1 (1.09)	
Moderate to severe tobacco dependence	117 (15.33)	79 (67.52)	14 (11.97)	11 (9.40)	7 (5.98)	6 (5.13)	
Relationship with patients							13.01
Spousal	390 (51.11)	286 (73.33)	39 (10.00)	36 (9.23)	22 (5.64)	7 (1.79)	
The patient’s child	61 (7.99)	49 (80.33)	4 (6.56)	5 (8.20)	2 (3.28)	1 (1.64)	
The patient’s parent	207 (27.13)	146 (70.53)	23 (11.11)	18 (8.70)	19 (9.18)	1 (0.48)	
Others^a^	105 (13.76)	86 (81.90)	10 (9.52)	5 (4.76)	4 (3.81)	0 (0.00)	
Gender							4.11
Male	485 (63.56)	357 (73.61)	49 (10.10)	44 (9.07)	27 (5.57)	8 (1.65)	
Female	278 (36.44)	210 (75.54)	27 (9.71)	20 (7.19)	20 (7.19)	1 (0.36)	
Marital Status							3.73
Others^b^	98 (12.84)	76 (77.55)	12 (12.24)	5 (5.10)	5 (5.10)	0 (0.00)	
Married	665 (87.16)	491 (73.83)	64 (9.62)	59 (8.87)	42 (6.32)	9 (1.35)	
Age (years)	59.86 ± 12.62	59.51 ± 13.06	61.21 ± 11.01	58.78 ± 11.25	63.02 ± 11.97	62.00 ± 8.08	1.25
Educational Level							7.67
Illiterate or semi-illiterate	223 (29.23)	159 (71.30)	23 (10.31)	18 (8.07)	19 (8.52)	4 (1.79)	
Primary school	224 (29.36)	169 (75.45)	21 (9.38)	21 (9.38)	10 (4.46)	3 (1.34)	
Junior high school	252 (33.03)	188 (74.60)	27 (10.71)	20 (7.94)	16 (6.35)	1 (0.40)	
High school and above	64 (8.39)	51 (79.69)	5 (7.81)	5 (7.81)	2 (3.12)	1 (1.56)	
Occupation							18.35
Farmer	442 (57.93)	323 (73.08)	45 (10.18)	44 (9.95)	24 (5.43)	6 (1.36)	
Workplace professionals and students	61 (7.99)	46 (75.41)	11 (18.03)	3 (4.92)	1 (1.64)	0 (0.00)	
Unemployed	190 (24.90)	139 (73.16)	16 (8.42)	14 (7.37)	18 (9.47)	3 (1.58)	
Others^c^	70 (9.17)	59 (84.29)	4 (5.71)	3 (4.29)	4 (5.71)	0 (0.00)	
Religious Belief							7.09
No	681 (89.25)	512 (75.18)	67 (9.84)	51 (7.49)	43 (6.31)	8 (1.17)	
Yes	82 (10.75)	55 (67.07)	9 (10.98)	13 (15.85)	4 (4.88)	1 (1.22)	
Annual Household Income^d^							22.95*
Level 1	261 (34.21)	207 (79.31)	15 (5.75)	20 (7.66)	15 (5.75)	4 (1.53)	
Level 2	163 (21.36)	114 (69.94)	17 (10.43)	17 (10.43)	12 (7.36)	3 (1.84)	
Level 3	158 (20.71)	110 (69.62)	28 (17.72)	14 (8.86)	6 (3.80)	0 (0.00)	
Level 4	181 (23.72)	136 (75.14)	16 (8.84)	13 (7.18)	14 (7.74)	2 (1.10)	
Caregiving Duration							13.73
<1 year	17 (2.23)	13 (76.47)	1 (5.88)	3 (17.65)	0 (0.00)	0 (0.00)	
1–2 year	4 (0.52)	2 (50.00)	0 (0.00)	2 (50.00)	0 (0.00)	0 (0.00)	
2–3 year	92 (12.06)	67 (72.83)	8 (8.70)	9 (9.78)	7 (7.61)	1 (1.09)	
>3 year	650 (85.19)	485 (74.62)	67 (10.31)	50 (7.69)	40 (6.15)	8 (1.23)	
Daily Average Caregiving Hours, hours	5.13 ± 4.91	4.76 ± 4.52	5.38 ± 5.21	5.94 ± 5.45	8.15 ± 6.62	4.22 ± 6.22	5.94***
Financial Assistance							12.16*
No	392 (51.38)	307 (78.32)	40 (10.20)	26 (6.63)	17 (4.34)	2 (0.51)	
Yes	371 (48.62)	260 (70.08)	36 (9.70)	38 (10.24)	30 (8.09)	7 (1.89)	
Total Household Debt (per 10 thousand RMB)	2.33 ± 7.93	2.01 ± 6.69	5.16 ± 16.21	2.14 ± 4.22	1.94 ± 4.86	1.74 ± 2.92	2.72*

*Note:* BPRS, Brief Psychiatric Rating Scale.

a Including the patient’s grandparents, maternal grandparents, relatives or friends.

b Including never married, divorced and widowed.

c Including retirement and engaging in informal occupations.

d Divided into levels 1 to 4 based on quartile scores, with higher levels indicating higher income. Due to rounding, the sum of some percentage values may not equal 100% (e.g. 99.99%).

*p<0.05, ***p<0.001.

Table 2 shows caregiver characteristics and physical abuse frequency. A total of 114 caregivers (14.9%) reported physical abuse. Of these, one caregiver (0.1%) abused the patient more than once per day, 14 (1.8%) more than once per week, 33 (4.3%) more than once per month, and 66 (8.7%) more than once per year. Higher BPRS scores in patients were significantly associated with greater frequency of physical abuse (*p* < 0.001). Annual household income and average daily caregiving time were also significantly associated with physical abuse frequency (both *p* < 0.05).

**Table 2.** Frequency of caregiver physical abuse toward SMDs patients (n (%)/mean ± SD)

Variables		Frequency of physical abuse by caregivers toward patients					*F*/*χ^2^*
	Total	Never	Several times a year (at least once, but less than once a month)	Several times a month (at least once, but less than once a week)	Several times a week (at least once, but less than once a day)	Once a day or more	
Total	763 (100.00)	649 (85.06)	66 (8.65)	33 (4.32)	14 (1.83)	1 (0.13)	
BPRS score	51.09 ± 20.48	50.31 ± 20.24	50.71 ± 19.67	58.91 ± 22.94	66.93 ± 16.79	102.00 ± 0.00	5.20***
Tobacco dependence							13.39
Non-smoking	554 (72.61)	480 (86.64)	45 (8.12)	21 (3.79)	8 (1.44)	0 (0.00)	
Mild tobacco dependence	92 (12.06)	79 (85.87)	8 (8.70)	3 (3.26)	2 (2.17)	0 (0.00)	
Moderate to severe tobacco dependence	117 (15.33)	90 (76.92)	13 (11.11)	9 (7.69)	4 (3.42)	1 (0.85)	
Relationship with patients							9.69
Spousal	390 (51.11)	328 (84.10)	32 (8.21)	19 (4.87)	10 (2.56)	1 (0.26)	
The patient’s child	61 (8.00)	56 (91.80)	4 (6.56)	1 (1.64)	0 (0.00)	0 (0.00)	
The patient’s parent	207 (27.13)	173 (83.57)	19 (9.18)	11 (5.31)	4 (1.93)	0 (0.00)	
Others^a^	105 (13.76)	92 (87.62)	11 (10.48)	2 (1.90)	0 (0.00)	0 (0.00)	
Gender							1.28
Male	485 (63.56)	410 (84.54)	44 (9.07)	22 (4.54)	8 (1.65)	1 (0.21)	
Female	278 (36.44)	239 (85.97)	22 (7.91)	11 (3.96)	6 (2.16)	0 (0.00)	
Marital Status							3.09
Others^b^	98 (12.84)	83 (84.69)	11 (11.22)	4 (4.08)	0 (0.00)	0 (0.00)	
Married	665 (87.16)	566 (85.11)	55 (8.27)	29 (4.36)	14 (2.11)	1 (0.15)	
Age (years)	59.86 ± 12.62	59.56 ± 13.06	61.59 ± 10.03	61.18 ± 8.87	61.93 ± 10.69	69.00 ± 0.00	0.71
Educational Level							8.60
Illiterate or semi-illiterate	223 (29.23)	179 (80.27)	24 (10.76)	14 (6.28)	5 (2.24)	1 (0.45)	
Primary school	224 (29.36)	193 (86.16)	18 (8.04)	9 (4.02)	4 (1.79)	0 (0.00)	
Junior high school	252 (33.03)	222 (88.10)	18 (7.14)	8 (3.17)	4 (1.59)	0 (0.00)	
High school and above	64 (8.39)	55 (85.94)	6 (9.38)	2 (3.12)	1 (1.56)	0 (0.00)	
Occupation							15.54
Farmer	442 (57.93)	366 (82.81)	44 (9.95)	23 (5.20)	8 (1.81)	1 (0.23)	
Workplace professionals and students	61 (7.99)	52 (85.25)	8 (13.11)	1 (1.64)	0 (0.00)	0 (0.00)	
Unemployed	190 (24.90)	164 (86.32)	12 (6.32)	8 (4.21)	6 (3.16)	0 (0.00)	
Others^c^	70 (9.17)	67 (95.71)	2 (2.86)	1 (1.43)	0 (0.00)	0 (0.00)	
Religious Belief							1.66
No	681 (89.25)	579 (85.02)	61 (8.96)	28 (4.11)	12 (1.76)	1 (0.15)	
Yes	82 (10.75)	70 (85.37)	5 (6.10)	5 (6.10)	2 (2.44)	0 (0.00)	
Annual Household Income^d^							27.10**
Level 1	261 (34.21)	232 (88.89)	14 (5.36)	12 (4.59)	2 (0.77)	1 (0.38)	
Level 2	163 (21.36)	131 (80.37)	15 (9.20)	9 (5.52)	8 (4.91)	0 (0.00)	
Level 3	158 (20.71)	129 (81.65)	23 (14.56)	6 (3.79)	0 (0.00)	0 (0.00)	
Level 4	181 (23.72)	157 (86.74)	14 (7.74)	6 (3.31)	4 (2.21)	0 (0.00)	
Caregiving Duration							4.10
<1 year	17 (2.23)	14 (82.35)	2 (11.76)	1 (5.88)	0 (0.00)	0 (0.00)	
1–2 year	4 (0.52)	3 (75.00)	1 (25.00)	0 (0.00)	0 (0.00)	0 (0.00)	
2–3 year	92 (12.06)	75 (81.52)	9 (9.78)	5 (5.43)	3 (3.26)	0 (0.00)	
>3 year	650 (85.19)	557 (85.69)	54 (8.31)	27 (4.15)	11 (1.69)	1 (0.15)	
Daily Average Caregiving Hours, hours	5.13 ± 4.91	4.91 ± 4.84	6.68 ± 5.39	6.27 ± 5.33	5.29 ± 3.22	1.00 ± 0.00	2.62*
Financial Assistance							7.07
No	392 (51.38)	344 (87.76)	31 (7.91)	13 (3.32)	4 (1.02)	0 (0.00)	
Yes	371 (48.62)	305 (82.21)	35 (9.43)	20 (5.39)	10 (2.69)	1 (0.27)	
Total Household Debt (per 10 thousand RMB)	2.33 ± 7.93	2.09 ± 6.65	4.89 ± 16.76	2.69 ± 4.04	0.64 ± 1.39	0.00 ± 0.00	2.08

*Note:* BPRS, Brief Psychiatric Rating Scale.

a Including the patient’s grandparents, maternal grandparents, relatives or friends.

b Including never married, divorced and widowed.

c Including retirement and engaging in informal occupations.

d Divided into levels 1 to 4 based on quartile scores, with higher levels indicating higher income. Due to rounding, the sum of some percentage values may not equal 100% (e.g. 99.99%).

*p<0.05, **p<0.01, ***p<0.001.

### Associations between psychiatric symptom severity of SMDs patients, tobacco dependence of caregivers and abusive behaviors of caregivers

The ordered logistic regression results are shown in Table 3. Psychiatric symptom severity (BPRS scores) was significantly and positively associated with caregiver abuse. Each one-point increase in BPRS score was associated with a 1.8% higher likelihood of more frequent verbal abuse (OR = 1.018, 95% CI: 1.010–1.026; *p* < 0.001) and a 1.5% higher likelihood of more frequent physical abuse (OR = 1.015, 95% CI: 1.005–1.025; *p* = 0.004).

**Table 3.** Associations between psychiatric symptom severity of SMDs patients, tobacco dependence of caregivers and abusive behaviors of caregivers

Variables	Frequency of verbal abuse by caregivers toward patients			Frequency of physical abuse by caregivers toward patients		
	OR	95% CI	*p* value	OR	95% CI	*p* value
BPRS score	1.018	(1.010, 1.026)	<0.001	1.015	(1.005, 1.025)	0.004
Tobacco dependence						
Non-smoking	1(Ref.)			1(Ref.)		
Mild tobacco dependence	1.797	(1.073, 3.010)	0.026	1.100	(0.547, 2.212)	0.790
Moderate to severe tobacco dependence	1.851	(1.136, 3.016)	0.013	2.292	(1.287, 4.079)	0.005
Relationship with patients						
Spousal	1(Ref.)			1(Ref.)		
The patient’s child	0.714	(0.341, 1.494)	0.371	0.493	(0.176, 1.382)	0.179
The patient’s parent	1.188	(0.770, 1.833)	0.437	0.946	(0.549, 1.632)	0.842
Others^a^	0.692	(0.384, 1.246)	0.220	0.787	(0.388, 1.597)	0.507
Gender						
Male	1(Ref.)			1(Ref.)		
Female	0.994	(0.654, 1.509)	0.976	0.939	(0.559, 1.580)	0.814
Marital Status						
Others^b^	1(Ref.)			1(Ref.)		
Married	1.593	(0.881, 2.878)	0.123	1.039	(0.515, 2.093)	0.915
Age (years)	1.002	(0.984, 1.019)	0.864	1.004	(0.982, 1.027)	0.732
Educational Level						
Illiterate or semi-illiterate	1(Ref.)			1(Ref.)		
Primary school	0.662	(0.418, 1.049)	0.079	0.512	(0.293, 0.895)	0.019
Junior high school	0.746	(0.460, 1.209)	0.234	0.455	(0.249, 0.831)	0.010
High school and above	0.600	(0.287, 1.255)	0.175	0.626	(0.265, 1.483)	0.287
Occupation						
Farmer	1(Ref.)			1(Ref.)		
Workplace professionals and students	1.120	(0.567, 2.210)	0.744	1.014	(0.434, 2.370)	0.975
Unemployed	0.800	(0.510, 1.255)	0.332	0.722	(0.411, 1.269)	0.257
Others^c^	0.482	(0.233, 0.998)	0.049	0.233	(0.069, 0.788)	0.019
Religious Belief						
No	1(Ref.)			1(Ref.)		
Yes	1.700	(1.009, 2.863)	0.046	1.246	(0.619, 2.507)	0.538
Annual Household Income^d^						
Level 1	1(Ref.)			1(Ref.)		
Level 2	1.805	(1.126, 2.895)	0.014	2.393	(1.339, 4.275)	0.003
Level 3	1.729	(1.064, 2.809)	0.027	2.168	(1.178, 3.993)	0.013
Level 4	2.001	(1.178, 3.399)	0.010	1.992	(1.005, 3.948)	0.048
Caregiving Duration						
<1 year	1(Ref.)			1(Ref.)		
1–2 year	2.479	(0.274, 22.430)	0.419	1.692	(0.103, 27.736)	0.712
2–3 year	0.911	(0.257, 3.233)	0.886	0.841	(0.199, 3.565)	0.815
>3 year	0.849	(0.259, 2.789)	0.788	0.662	(0.170, 2.573)	0.551
Daily Average Caregiving Hours, hours	1.067	(1.033, 1.103)	<0.001	1.052	(1.012, 1.095)	0.011
Financial Assistance						
No	1(Ref.)			1(Ref.)		
Yes	1.409	(0.996, 1.995)	0.053	1.449	(0.941, 2.232)	0.092
Total Household Debt (per 10 thousand RMB)	1.010	(0.993, 1.028)	0.243	1.015	(0.996, 1.035)	0.132

*Note:* BPRS, Brief Psychiatric Rating Scale; CI: confidence interval; OR, odds ratios.

a Including the patient’s grandparents, maternal grandparents, relatives or friends.

b Including never married, divorced and widowed.

c Including retirement and engaging in informal occupations.

d Divided into levels 1 to 4 based on quartile scores, with higher levels indicating higher income.

Caregiver tobacco dependence was also a significant predictor. Compared with non-smokers, caregivers with mild dependence were more likely to commit verbal abuse (OR = 1.797, 95% CI: 1.073–3.010; *p* = 0.026), while those with moderate-to-severe dependence had an 85.1% higher likelihood (OR = 1.851, 95% CI: 1.136–3.016; *p* = 0.013). For physical abuse, caregivers with moderate-to-severe dependence had more than twice the odds of abuse compared with non-smokers (OR = 2.292, 95% CI: 1.287–4.079; *p* = 0.005), whereas mild dependence was not significantly different from non-smoking (OR = 1.100; *p* = 0.790).

In addition, higher annual household income (*p* < 0.05) and longer daily caregiving duration (*p* < 0.05) were significantly associated with increased frequency of both forms of abuse. By contrast, caregivers with primary or junior middle school education (vs. illiterate/semi-literate; *p* < 0.05) and those in other occupations (*p* < 0.05) were less likely to commit physical abuse.

### Moderating effect of tobacco dependence

Table 4 presents the moderation analysis. Caregiver tobacco dependence significantly moderated the association between patient psychiatric symptom severity and verbal abuse frequency. Compared with non-smokers, caregivers with moderate-to-severe dependence showed a stronger association: each one-point increase in BPRS score was associated with a 2.4% higher likelihood of more frequent verbal abuse (OR = 1.024, 95% CI: 1.002–1.046; *p* = 0.031). The moderating effect was not significant for mild dependence (*p* = 0.496).

**Table 4.** Moderating effect of tobacco dependence on the association between psychiatric symptom severity and caregiver abuse frequency

Items	Frequency of verbal abuse by caregivers toward patients			Frequency of physical abuse by caregivers toward patients		
	OR	95% CI	*p* value	OR	95% CI	*p* value
BPRS score	1.012	(1.002, 1.022)	0.018	1.015	(1.002, 1.027)	0.025
Tobacco dependence						
Non-smoking	1 (Ref.)			1 (Ref.)		
Mild tobacco dependence	1.158	(0.300, 4.477)	0.831	0.966	(0.158, 5.913)	0.970
Moderate to severe tobacco dependence	0.499	(0.135, 1.846)	0.298	2.242	(0.538, 9.344)	0.268
BPRS score × Non-smoking	1 (Ref.)			1 (Ref.)		
BPRS score × Mild tobacco dependence	1.008	(0.985, 1.032)	0.496	1.002	(0.973, 1.033)	0.879
BPRS score × Moderate to severe tobacco dependence	1.024	(1.002, 1.046)	0.031	1.000	(0.977, 1.024)	0.975

*Note:* BPRS, Brief Psychiatric Rating Scale; CI: confidence interval; OR, odds ratios.

**Figure 1.**
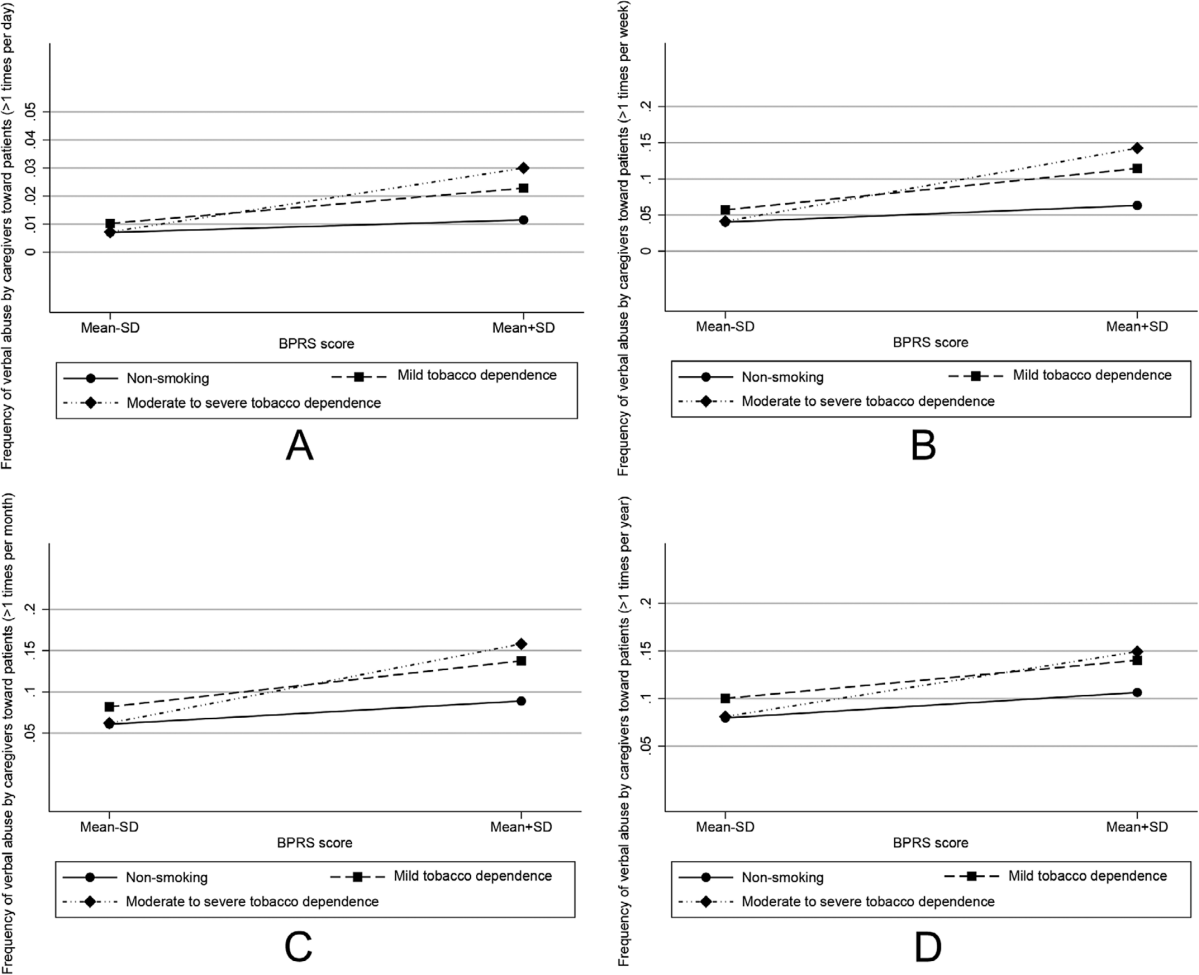
The predicted probability of verbal abuse across BPRS scores, stratified by tobacco dependence level. A, Frequency of verbal abuse: once a day or more versus never. B, Frequency of verbal abuse: several times a week versus never. C, Frequency of verbal abuse: several times a month versus never. D, Frequency of verbal abuse: several times a year versus never.

No significant moderating effect of tobacco dependence was observed for physical abuse (all *p* > 0.05). Figure 1 illustrates the predicted probability of verbal abuse across BPRS scores, stratified by tobacco dependence level, showing patterns across different abuse frequencies: Once a day or more (panel A), Several times a week (panel B), Several times a month (panel C), and more than once per year (panel D). All four panels (A, B, C, and D) indicate that the association between BPRS scores and the frequency of verbal abuse is stronger among caregivers with moderate-to-severe tobacco dependence than among non-smoking caregivers.

## Discussion

Based on paired data from 763 patients with SMDs and their family caregivers in rural China, we found that psychiatric symptom severity was significantly associated with the frequency of both verbal and physical abuse by caregivers. Moreover, moderate-to-severe tobacco dependence among caregivers was not only directly associated with a higher risk of abuse but also significantly strengthened the association between psychiatric symptom severity and verbal abuse. To our knowledge, this is the first study in rural China to demonstrate both the association between psychiatric symptom severity and caregiver abuse, and the moderating role of tobacco dependence, thereby providing empirical grounds for family-based interventions and public health strategies.

Few studies have specifically assessed caregiver-perpetrated abuse of SMD patients in China, and available findings vary by region and study design. Li et al. reported a prevalence of verbal abuse ranging from 15.9% to 21.5% and physical abuse of 4.2% among caregivers of schizophrenia patients in southern China (Li et al., 2023). Wang et al. (2020) found that 18.9% of rural patients experienced at least one violent incident within 6 months, though the perpetrators were not specified. By contrast, our study documented higher prevalence rates in rural areas, with 25.7% for verbal abuse and 14.9% for physical abuse, suggesting that caregiver abuse may be particularly concerning in these settings.

International comparisons indicate that the prevalence of abuse in rural China is at an intermediate level globally. In Ethiopia, 36.3% of patients with SMDs reported caregiver violence, without differentiation of abuse types (Wale et al., 2025). In India, 39% of patients experienced physical abuse by family members, and the overall prevalence of any abuse reached 72% (Bhatia et al., 2016). In Israel, 66% of patients reported verbal abuse within a year, most commonly by friends and parents (Karni-Vizer & Salzer, 2016). A systematic review further showed wide variation, with the prevalence of family violence against SMDs patients ranging from 14.8% to 95.2% in men and 10% to 67% in women(Rani et al., 2022), showing significant variations. In rural areas of China, the causes of abuse may be linked to caregivers’ limited understanding of mental illnesses (Li & Reavley, 2021). They often misinterpret the patients’ symptoms as intentional behavior or moral issues, thereby rationalizing their abusive actions. Deep-rooted Confucian familism, which emphasizes family responsibility and regards caregiving as a private duty (Yang, 2004), further reinforces the invisibility of abuse by limiting external oversight and intervention.

Our finding that psychiatric symptom severity was associated with caregiver abuse is consistent with prior evidence in dementia research (Fang et al., 2021), but evidence among SMDs patients remains scarce. This study extends the literature by demonstrating that psychiatric symptom severity is not only associated with poor clinical outcomes but also linked to a higher risk of abuse within family settings. Mechanisms are likely multifaceted. Psychologically, worsening symptoms heighten both the emotional and physical caregiving burden, increasing frustration and hostility in unsupported caregivers (Kochhar et al., 2024). Within the stress–coping framework, persistent symptoms act as chronic stressors; in the absence of adaptive coping and social support, caregivers may resort to abusive behaviors as maladaptive outlets (Pearlin et al., 1990; Pickering et al., 2018; Umer et al., 2025). Sociologically, patients’ behavioral symptoms can disrupt family roles, dynamics, and power structures (Kochhar et al., 2024; Labrum & Solomon, 2015; Lin & Giles, 2013), prompting caregivers to use verbal or physical force in an attempt to regain control (Lin & Giles, 2013; Pillemer & Finkelhor, 1989). Biologically, chronic exposure to severe psychiatric symptoms may dysregulate caregivers’ hypothalamic–pituitary–adrenal axis and promote systemic inflammation (Bamber et al., 2023; Beadle & Jain, 2021), diminishing emotional regulation and increasing aggressive reactivity. Taken together, these perspectives suggest that stress, strain, and self-control processes jointly shape the mechanisms underlying caregiver abuse. Within the stress–coping framework (Biggs et al., 2017), persistent patient symptoms act as chronic stressors that erode caregivers’ coping resources. As strain theory posits (Zhang et al., 2011; Zhang, 2019), accumulated frustration and perceived caregiving failure generate psychological tension, which caregivers may attempt to relieve through smoking or aggressive expression. Concurrently, the self-control perspective (Gottfredson & Hirschi, 1990) highlights that nicotine dependence compromises emotional regulation and inhibitory capacity, thereby lowering the threshold for impulsive or abusive reactions. This integrated model suggests that caregiver abuse represents not only a behavioral manifestation of stress overload but also a consequence of impaired self-regulation under chronic strain.

We also observed that moderate-to-severe tobacco dependence significantly moderated the association between psychiatric symptom severity and verbal abuse. Physiologically, nicotine dependence reshapes the brain’s reward and stress-regulation systems, rendering dependent individuals more vulnerable to mood fluctuations and impulsivity during nicotine depletion (Bruijnzeel, 2012). Nicotine withdrawal is also associated with cognitive deficits – including impairments in attention, working memory, and inhibitory control – that compromise caregivers’ ability to regulate responses to patients’ symptoms (Ashare et al., 2014). Furthermore, smokers display marked deficits in inhibitory control under tobacco-related cues (Hou et al., 2024), suggesting that caregivers with nicotine dependence may be more prone to loss of control when psychiatric symptoms worsen. At the behavioral level, while smoking is often perceived by caregivers and health professionals as a coping strategy for stress and severe psychiatric symptoms, such beliefs sustain smoking culture (Cookson et al., 2014; Sheals et al., 2016). In reality, the short-term relief derives largely from alleviation of withdrawal symptoms, while the long-term effects of smoking are detrimental to brain and mental health (Taylor et al., 2021; Taylor & Treur, 2023). Compared with non-dependent caregivers, those with moderate-to-severe dependence are more vulnerable within the stress–coping balance (Bruijnzeel, 2012; Pietras et al., 2011); even minor symptom exacerbations may exceed their tolerance thresholds, substantially elevating the risk of verbal abuse. Conceptually, we treated tobacco dependence as a moderator rather than a mediator, given that it represents a relatively stable tendency shaping caregivers’ emotional and behavioral responses to stress. Within the stress-coping and self-control frameworks, tobacco dependence may alter the strength of the association between patients’ psychiatric symptom severity and abusive behaviors by lowering caregivers’ inhibitory control and emotional regulation thresholds. This conceptualization aligns with moderation logic, which focuses on effect modification rather than causal transmission.

This study highlights the unique challenges of rural contexts, where mental health resources are scarce and caregivers often lack access to professional guidance, psychiatric support, respite care, or psychological assistance. Structural deficiencies leave caregivers isolated and solely responsible for intensive caregiving, contributing to exhaustion and burnout (Adelman et al., 2014; Hu et al., 2023). Economic fragility further compounds this burden: medical costs, loss of household labor, and time constraints collectively increase strain. Interestingly, we found higher abuse risk among households with greater annual income, possibly reflecting heightened sensitivity to stigma and reputational concerns, which intensify psychological stress (Wang et al., 2023; Yin et al., 2020). Longer daily caregiving hours also directly erode physical and emotional reserves, lowering thresholds for emotional regulation and elevating risk of abusive responses (Adelman et al., 2014).

These findings offer practical implications for public health strategies, particularly within the context of rural China. First, caregiver-centered interventions, such as family education programs (Farhall et al., 2020), psychoeducation, stress management, and behavioral counseling, should be systematically integrated into primary mental health services to enhance caregivers’ knowledge, emotional regulation, and coping capacity. The existing community-based service network, which includes the family doctor contract service and basic public health programs, provides a feasible delivery platform: family doctors and community health workers can screen caregivers for tobacco dependence, provide brief advice, offer follow-up support, and link dependent caregivers to specialized cessation or mental health services. Second, tobacco control should be prioritized within mental health care and primary care for caregivers. Evidence-based smoking-cessation strategies that combine behavioral counseling, such as cognitive behavioral therapy (Malbos et al., 2023), pharmacotherapy where appropriate, and sustained follow-up improve quit rates and can mitigate nicotine-related emotional dysregulation that increases the risk of abusive responses. Training primary-care teams and family doctors to deliver brief interventions and establishing referral pathways to more intensive cessation services would be pragmatic steps in resource-limited rural settings. Community-based navigators and outreach programs have been shown to increase uptake of cessation treatments among disadvantaged smokers and may be adapted to reach caregiving populations. Third, formalized community-based peer support and mutual-aid networks deserve strengthening as complementary strategies. Peer groups, caregiver support programs, and community mental-health interventions can reduce isolation, normalize help-seeking, and provide practical coping skills that decrease reliance on maladaptive behaviors such as smoking; interventions targeting caregivers in low- and middle-income countries have demonstrated medium-to-large effects on caregiver outcomes (perceived burden, depression, and anxiety) and are therefore promising models for scale-up (Chen et al., 2025). Embedding peer support within the family-doctor and community-health frameworks can create low-cost, sustainable platforms for ongoing psychosocial support. Finally, cross-sectoral policy coordination is essential: aligning mental health, tobacco control, primary care, and social welfare policies will create a more supportive environment for both patients and caregivers. In the long run, addressing caregiver abuse requires a coordinated, system-level response. Given the documented global burden of household violence and the elevated risk of abuse among people with severe mental disorders, caregiver-focused interventions that combine addiction treatment, psychosocial support, and routine risk screening could reduce family violence and improve patient outcomes at a population scale. Although developed within the context of China’s primary-care reforms, these strategies may also inform similar efforts in other low- and middle-income countries.

This study has several strengths. The use of paired data from both patients and caregivers enhanced validity and explanatory power. The innovative inclusion of tobacco dependence as a moderator provided novel insights into mechanisms of abuse. Nonetheless, limitations should be acknowledged. First, as this is a cross-sectional study, the findings represent associations rather than established causal relationships. We therefore cannot fully exclude the possibility that caregivers’ tobacco dependence may also act as a mediating factor between psychiatric symptom severity and abusive behaviors. For instance, more severe psychiatric symptoms could heighten caregiving stress, which in turn increases smoking severity and subsequently elevates the risk of abuse. Future longitudinal or interventional studies are warranted to establish the temporal sequence and to test this potential mediating pathway through causal mediation analyses. Second, our measurement of abusive behaviors relied primarily on patient or relative reports and, in most cases, patients and their primary caregivers were interviewed in the same setting with a family doctor present to facilitate understanding and ensure safety. Although this procedure was necessary, given the communication difficulties and safety concerns inherent in interviewing some patients with severe mental disorders, it may have introduced social desirability and reporting biases. Caregivers might underreport abusive acts because of shame or stigma, while patients’ accounts could be affected by symptom severity or by caregivers’ presence during interviews. These factors may together lead to an underestimation of the true prevalence of abuse. To mitigate these issues, future studies should implement triangulated, multimodal assessment strategies. This approach would leverage multiple informants and methods, including separate patient interviews, caregiver reports, standardized assessments, and ethical observational methods, to significantly enhance measurement validity and reliability. Moreover, future research should also examine relatively stable and temporally antecedent background factors, such as caregivers’ educational attainment and socioeconomic status, as potential moderators of the relationship between patient symptoms and abusive behaviors. These characteristics are typically established prior to the caregiving context and remain stable over time, thus offering a clearer and more theoretically grounded understanding of effect modification across sociodemographic strata. Finally, because the majority of caregivers in our sample were family members (spouses, children, or parents), our findings may not fully capture the experiences and motivations of non-family caregivers, whose emotional involvement and caregiving roles may differ; future work should therefore explicitly compare family versus non-family caregivers. Despite these limitations, our study contributes new evidence on the interplay between psychiatric symptom severity and caregiver abuse in rural Chinese families and, importantly, identifies tobacco dependence as a significant moderator. These findings provide fresh perspectives for family interventions and public health strategies.

## Conclusion

In rural China, psychiatric symptom severity among SMDs patients is significantly associated with both verbal and physical abuse by family caregivers, and moderate-to-severe tobacco dependence further strengthens the association between psychiatric symptom severity and verbal abuse. Abuse thus reflects the cumulative influence of patient severity and caregiver dependence. Prevention and intervention strategies should therefore prioritize not only symptom management for patients but also psychological support and tobacco cessation interventions for caregivers, particularly in rural settings with limited mental health resources.

## Data Availability

The datasets generated and analyzed during the current study are not publicly available due to participant privacy and ethical restrictions, but are available from the corresponding author on reasonable request.
