# Is tobacco dependence a moderator of psychiatric symptom severity and caregiver abuse in rural families of patients with severe mental disorders? – CORRIGENDUM

**DOI:** 10.1017/S003329172510295X

**Published:** 2025-12-29

**Authors:** Afei Qin, Meiqi Wang, Yazhuo Qi, Kaixian Wang, Zhen Wei, Long Sun

**Affiliations:** 1 Department of Social Medicine and Health Management, School of Public Health, Cheeloo College of Medicine, Shandong University, Jinan, China; 2 NHC Key Lab of Health Economics and Policy Research, Shandong University, Jinan, China; 3 Center for Health Management and Policy Research, Shandong University, Jinan, China; 4 School (Institute) of Mental Health and Psychological Sciences, Cheeloo College of Medicine, Shandong University, Jinan, China

When this article was originally published in Psychological Medicine it contained an error in the name of the author Kaixian Wang. This has been updated in the original article.

The authors apologise for this error.
